# Anal disorders in pregnant and postpartum women: epidemiological, diagnostic and therapeutic aspects in 10 maternities of Bamako in Mali

**DOI:** 10.11604/pamj.2024.47.66.36210

**Published:** 2024-02-14

**Authors:** Adégné Togo, Lassana Kanté, Abdoulmouinou Poudiougo, Amadou Traoré, Amadou Bocoum, Youssouf Traoré, Madiassa Konaté, Moussa Younoussa Dicko, Moussa Samaké, Bah Amadou, Hawa Touré, Laurent Abramowitz

**Affiliations:** 1 Department of Surgery, Teaching Hospital Gabriel Toure, University of Sciences, Techniques and Technologies of Bamako (USTTB)/Faculty of Medicine and Dentistry (FMOS), Bamako, Mali; 2 Department of Gynecology Teaching Hospital Gabriel Toure, University of Sciences, Techniques and Technologies of Bamako (USTTB)/Faculty of Medicine and Dentistry (FMOS), Bamako, Mali; 3 Department of Hepato-gastroenterology Teaching Hospital Gabriel Toure, University of Sciences, Techniques and Technologies of Bamako (USTTB)/Faculty of Medicine and Dentistry (FMOS), Bamako, Mali; 4 Department of Surgery Secondary Hospital Commune IV, University of Sciences, Techniques and Technologies of Bamako (USTTB)/Faculty of Medicine and Dentistry (FMOS), Bamako, Mali; 5 Department of Hepato-gastroenterology Teaching Hospital, Bichat, Paris, France

**Keywords:** Anal pathology, pregnant women, postpartum

## Abstract

Bowel transit disturbances favored by pregnancy and injuries during childbirth would be triggering or aggravating factors for anal pathologies. The objective of this work was to study the epidemiology, diagnosis, and treatment of anal pathologies during pregnancy and 6 weeks after delivery. We carried out a prospective, multi-centric, and analytical study in 10 obstetric units in Bamako from June 1^st^, 2019, to May 31^st^, 2020. After informed consent, we enrolled all first-trimester pregnant women admitted to the hospitals and who were followed up through the postpartum. We conducted a rectal examination in each participant and an anoscope in those with an anal symptom. Hemorrhoidal diseases were diagnosed in the case of external hemorrhoids (thrombosis or prolapse) or internal hemorrhoids. During the study period, we followed up 1,422 pregnant women and we found 38.4% (546) with anal pathologies (hemorrhoidal diseases in 13% (192), anal fissure in 10.5% (150) and anal incontinence in 8.6% (123). Risk factors for the hemorrhoidal disease were age of patient ≥30 years old aRR=5.77, 95% CI 4.57-7.34; p=0.000; a existence of chronic constipation aRR=2.61, 95% CI 1.98-3.44; p=0.000; newborn weight >3500 g aRR= 1.61, 95% CI 1.25-2.07; p=0.000 and fetal expulsion time >20 minutes aRR= 6.04, 95% CI 5.07-7.27; p=0.000. The clinical signs observed were constipation, anal pain, bleeding, and pruritus. The treatment was based on counseling on hygiene and diet, the use of laxatives, local topicals, and analgesics along perineal rehabilitation. Anal pathologies were common during pregnancy and 6 weeks after delivery. Pregnant women must be screened systematically for such pathologies. Early diagnostic and appropriate treatment would reduce serious complications.

## Introduction

While pregnancy and childbirth are generally considered happy life episodes in women, these events can be affected by anal conditions. A third of parturient women develop an anal lesion after childbirth. These are essentially hemorrhoid thrombosis and anal fissures resulting in either major discomfort or severe pain [1]. Bowel transit disturbances favored by pregnancy and injuries during childbirth would be triggering or aggravating factors for anal pathologies [2]. The diagnosis is mostly late and the management is often either inadequate or inappropriate.

In pregnant women, a study conducted in 2003 at the Bichat Hospital in Paris, France found in 165 pregnant women 7.9% of hemorrhoidal pathologies during the third trimester of pregnancy and 20% in the immediate postpartum [3]. MacArthur C *et al*. have considered that traumatic deliveries with forceps, an extended eviction time, and a large baby increased the risk of hemorrhoidal complications [4]. Immediate postpartum has been found to be the most suitable period for anal thrombosis with 20% of parturients versus 8% of women in the third trimester of pregnancy [5].

Anal pathology is probably underestimated in Africa due to modesty, the use of traditional medicine, neglect, and lack of information. In Mali, in 2006, Traore A *et al*. [6] found in seven years an annual frequency of 21.4% of hemorrhoidal diseases on all outpatient and inpatient visits in the General Surgery Department of University Hospital Gabriel Touré. To the best of our knowledge, we did not find any previous study on anal pathologies in pregnant women in Mali. We carried out this work with the following objectives: to determine the frequency of anal pathologies in pregnant and postpartum women; to identify the main risk factors for anal pathologies during pregnancy and postpartum; to evaluate the medico-surgical treatment of anal pathologies observed during pregnancy and postpartum.

## Methods

**Study design and setting:** this is a 12-month prospective, descriptive, and analytical study from June 1^st^, 2019, to May 31^st^, 2020. It was multicenter in the following ten (10) maternity in Bamako. The maternity was located in the six district hospitals (communes I, II, III, IV, V, and VI); the Hospital of Kalanban Koro, the Gabriel Touré University Hospital, the Point G University Hospital, and the Mother Child Hospital Luxembourg. In each study site, we identified a midwife, a resident, an obstetrician-gynecologist, and a skilled surgeon trained in proctology to carry out this work.

**Study population:** we recruited all pregnant women who met our inclusion criteria, admitted for prenatal consultation at our study sites. Inclusion criteria: consenting first-trimester pregnant women, admitted to the hospital and followed up during pregnancy and postpartum. Non-inclusion criteria: women who had the first prenatal consultation after the first trimester of pregnancy. Each pregnant woman underwent obstetrical and proctological examinations in the 1^st^ and 3^rd^ trimesters of pregnancy and in the postpartum: during the proctological clinical examination, each patient had an inspection with a rectal touch on a patient examination table in either genu-pectoral position or left lateral decubitus. An anoscope was performed on women who had any anal symptoms.

**Data collection:** data have been centralized at the study coordinator level. Epi info™ version 3.5.1 was used for data collection and analysis. Over nine (9) months, we registered 1,422 pregnant women in the 10 maternity hospitals that met the inclusion criteria and we followed them up from the 1^st^ trimester up to six (6) weeks after delivery.

**Definitions:** the diagnosis of hemorrhoidal diseases was made in case of external hemorrhoid (thrombosis or prolapse) or internal hemorrhoidal disease. Anal incontinence was defined by the notion of loss of stool or gas during interrogation. The diagnosis of anal fissure was made on anal inspection and rectal examination.

**Statical analysis:** Epi info™ version 3.5.1 was used for analysis. We used frequencies for description and did multivariable regression to identify the risk factors.

**Ethical considerations:** this study has been approved by the Scientific Committee at the Gabriel Touré University Hospital. We strictly observed the confidentiality and privacy of our study participants.

## Results

**Figure 1:**
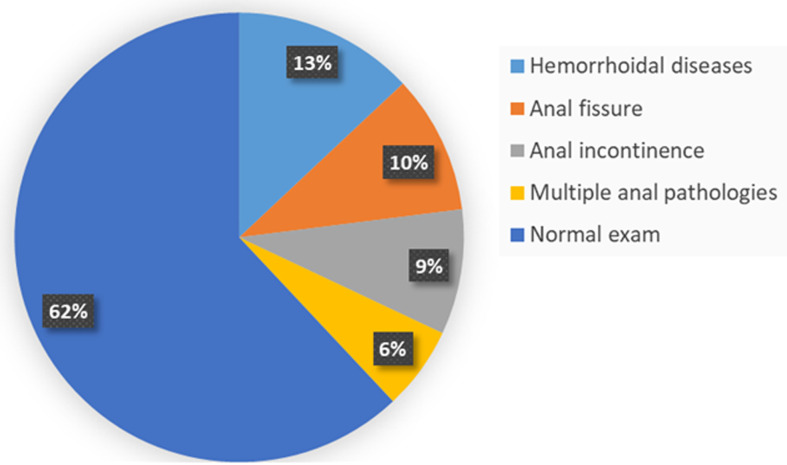
frequency of anal pathologies

**Frequency of anal pathologies:** we registered 1,422 pregnant women in the 10 maternities. We identified 38.4% (546) cases of anal pathology in pregnant women as followed 192 cases of hemorrhoidal diseases, 150 cases of anal fissures, 123 cases of anal incontinence, and 81 cases of multiple anal pathologies (Figure 1). We found an increase in the frequency of the 3 pathologies (anal fissure hemorrhoidal disease and anal incontinence) from the first trimester of pregnancy to the third trimester. This increase is more marked postpartum for anal fissure and anal incontinence (Table 1).

**Table 1:** recorded anal pathologies

Anal pathologies	Moment of diagnosis			Total
	First-trimester n (%)	Third-trimester n (%)	Post-partum n (%)	
Anal fissure	14(2.5)	33(6.1)	103(18.9)	150(27.5)
Hemorroidal disease	43(7.9)	72(13.2)	77(14.1)	192(35.2)
Anal Incontinence	25(4.6)	36(6.6)	62(11.3)	123(22.5)
Multiple anal pathologies	13(2.4)	26(4.7)	42(7.7)	81(14.8)
Total	95(17.4)	167(30.6)	284(52)	546(100)

**Clinical aspects of anal pathologies:** the average age of women was 26 years SD = 6 minimum 14 years and maximum 45 years. Upon examination of the patients, the signs found were constipation, anal bleeding, loss of gaze and Stoll, anal etching, and anal pain (Table 2, Table 3).

**Table 2:** clinical signs on examination of the 1422 pregnant women according to the period of pregnancy

Clinical signs	First-trimester n (%)	Third-trimester n (%)	Post-partum n (%)
Constipation	426(30)	820(57.6)	215(15.1)
Hemorrhoidal marked	150(10.5)	154(10.8)	190(13.4)
Anal pain	109(7.6)	185(13)	314(22.1)
Anal pruritus	79(5.5)	91(6.4)	142(10)
Rectal bleeding	18(1.2)	85(6)	143(10.1)
Fissure wound	23(1.6)	61(4.3)	145(10.2)
Loss of gas	29(2)	39(2.7)	69(4.9)
Loss of stool	5(0.3)	12(0.8)	18(1.3)
External hemorrhoidal package	12(0.8)	47(3.3)	85(6)

**Table 3:** frequency of signs reported by the 546 pregnant women with anal disease

Clinical signs	Anal pathologies		
	Hemorrhoidal disease n=255 (%)	Anal fissure n=165 (%)	Anal Incontinence n=159 (%)
Constipation	197(77.3)	147(89.1)	65(40.9)
Diarrhea	15(5.8)	6(3.6)	12(7.5)
Anal pain	135(52.9)	152(92.1)	5(3.1)
Anal pruritus	80(31.4)	73(44.2)	23(14.4)
Rectal bleeding	55(21.5)	78(47.3)	4(2.5)
Anal oozing	37(14.5)	15(9.1)	7(4.4)

Risk factors for anal pathologies are variable (Table 4, Table 5, Table 6). For hemorrhoidal disease the risk factors were age of patient ≥ 30 years old aRR=5.74, 95% CI 4.56-7.34; p=0.000; an existence of chronic constipation aRR=2.61, 95% CI 1.98-3.44; p=0.000; newborn weight >3500 g aRR= 1.61, 95% CI 1.25-2.07; p=0.000 and fetal expulsion time >20 minutes OR= 6.04, 95% CI 5.07-7.27; p=0.000. For anal incontinence, the risk factors were age of patient ≥ 30 years old aRR= 3.36, 95% CI 2.51-4.49. The body mass index >25 kg/m^2^ aRR= 4.81, 95% CI 3.64-6.49; p=0.000; multiparity aRR=2.14, 95% CI 1.46-3.12; p=0.003 and newborn weight >3500 g aRR= 4.11, 95% CI 3.11-5.44; p=0.000. For anal fissure, risk factors were the existence of chronic constipation aRR=5.99, 95% CI 3.71-9.60; p=0.000; newborn weight >3500 g aRR= 0.44, 95% CI 0.25-0.77; p=0.002 and fetal expulsion time >20 minutes aRR= 0.29, 95% CI 0.14-0.59; p=0.000.

**Table 4:** risk factors for hemorrhoidal disease

Variables		Hemorrhoidal diseases		
		Frequency n (%)	Adjusted RR (95% CI)	P-Value
Age (years)	≥ 30	175 (44.9)	5.74(4.56-7.34)	< 0.001
	< 30	80 (7.8)		
Parity	Multiparity	130 (16.9)	0.51(0.41-0.64)	< 0.001
	Primiparity	125 (32.5)		
Constipation	Yes	197 (24)	2.61(1.98-3.44)	< 0.001
	No	58 (9.6)		
Body mass index (BMI) (kg/m^2^)	≥ 25	90 (28,8)	1.93(1.54-2.41)	< 0.001
	< 25	165 (14.9)		
Childbirth	Low way	184 (15.3)	0.46(0.36-0.58)	< 0.001
	Cesarean	71 (32.7)		
Use of suction cup	Yes	20 (31.7)	1.54(1.05-2.02)	0.035
	No	235 (20.6)		
Duration of evictions (minutes)	> 20	130 (73.4)	6.04(5.01-7.27)	< 0.001
	≤ 20	125 (26.3)		
Newborn weight (g)	> 3500	60 (26.3)	1.61(1.25-2.07)	< 0.001
	≤ 3500	195 (16.3)		
Perineal tear	Yes	5 (5.2)	0.23(0.09-0.54)	< 0.001
	No	250 (22.5)		

**Table 5:** risk factors for anal fissure

Variables		Anal fissure		
		Frequency n (%)	Adjusted RR (95% CI)	P-Value
Age (years)	≥ 30	35 (9)	0.73(0.49-1.01)	0.057
	< 30	130 (12.6)		
Parity	Multiparity	87 (11.3)	0.55(0.42-0.73)	< 0.001
	Primiparity	78 (20.3)		
Constipation	Yes	147 (18)	5.99(3.71-9.60)	< 0.001
	No	18 (3)		
Body mass index (BMI) (kg/m^2^)	≥ 25	34 (10.9)	0.99(0.64-1.31)	0.620
	< 25	131 (11.8)		
Childbirth	Low way	113 (9.4)	0.42(0.31-0.57)	< 0.001
	Cesarean	48 (22.1)		
Use of suction cup	Yes	3 (4.8)	0.33(0.11-1.02)	0.034
	No	162 (14.2)		
Duration of evictions(minutes)	> 20	8 (4.5)	0.29(0.14-0.59)	< 0.001
	≤ 20	157 (15.3)		
Newborn weight (g)	> 3500	13 (5.7)	0.44(0.25-0.77)	0.002
	≤ 3500	152 (12.7)		
Perineal tear	Yes	7 (7.3)	0.51(0.24-1.05)	0.057
	No	158 (14.2)		

**Table 6:** risk factors for anal incontinence

Variables		Anal incontinence		
		Frequency n (%)	Adjusted RR (95% CI)	P-Value
Age (years)	≥ 30	175 (44.9)	3.36(2.5-4.49)	< 0.001
	< 30	80 (7.8)		
Parity	Multiparity	130 (16.9)	2.14(1.46-3.12)	0.003
	Primiparity	125 (32.5)		
Constipation	Yes	197 (24)	0.6(0.37-0.68)	< 0.001
	No	58 (9.6)		
BMI (kg/m^2^)	≥ 25	90 (28.8)	4.86(3.64-6.49)	< 0.001
	< 25	165 (14.9)		
Childbirth	Low way	184 (15.3)	0.88(0.59-1.29)	0.52
	Cesarean	71 (32.7)		
Use of suction cup	Yes	20 (31.7)	3.09(2.31-4.58)	< 0.001
	No	235 (20.6)		
Duration of evictions (minutes)	> 20	130 (73.4)	1.63(1.61-2.30)	0.005
	≤ 20	125 (26.3)		
Newborn weight (g)	> 3500	60 (26.3)	4.11(3.11-5.14)	< 0.001
	≤ 3500	195 (16.3)		
Perineal tear	Yes	5 (5.2)	0.77(0.42-1.42)	0.402
	No	250 (22.5)		

**Treatment:** the treatment was medical in all 546 pregnant women. Education for a soft stool diet was systematically given to each study participant. The consumption of foods rich in vegetable fiber was prescribed. A list of local foods containing these fibers was provided to patients. Among 1,422 pregnant women seen, transit regulator and laxative drugs were prescribed for 627 women (44%); analgesics were administered in 150 women (10.48%), and local topicals in 220 patients (15%). In postpartum, perineal rehabilitation was carried out in 28 patients.

## Discussion

**Frequencies:** the frequency of anal pathologies during pregnancy and postpartum has not been evaluated in Mali. This pathology, considered taboo, is rarely mentioned during prenatal consultations. We report a frequency of 38.4% (546) who have developed anal disease. This high frequency is also reported by other European authors Abramowitz *et al*. [7] and Poskus T *et al*. [8] respectively 44.4% and 43.9%. Ferdinande K *et al*. [9] found a higher frequency of 68.5%. And among the various anal pathologies mentioned, hemorrhoidal disease was the most frequent anal pathology at 18%. The same findings were made by Poskus T *et al*. [8]. Screening for these pathologies should be a standard feature of prenatal consultations.

**Risk factors:** in the literature, risk factors are known for the various anal pathologies. We have identified the following factors: Age ≥ 30 years, chronic constipation, multiparity, newborn weight >3500 g, body mass index ≥ 25 kg/m^2^, and fetal expulsion time of more than 20 minutes. Abramowitz L *et al*., Poskus T *et al*., and Mirhaidari *et al*. [7,8,10] have identified these same risk factors. Median episiotomy, obstetric anal sphincter injury, and forceps delivery may also aggravate anal incontinence [11,12].

**Clinical signs:** the signs that bring pregnant women to the doctor are very annoying and sometimes worrying. We have identified anal pain, rectal bleeding, anal pruritus, and loss of stool and gas as clinical signs. In their study by Poskus T *et al*., Herold A *et al*. and Ollende *et al*. [8,13,14] identified bleeding and pain as signs of anal pathology. Pruritus usually occurs secondary to hemorrhoidal prolapsus [15]. Anal incontinence is a real handicap in 21^st^-century life. Loss of gas and/or stool has been reported by many of our patients. Other authors Alonso-Coello P *et al*., Ferdinande *et al*., and Freymond *et al*. [9,16,17] found 7.9% anal incontinence postpartum.

**Treatment:** treatment of anal pathologies in pregnant women must take into account the compatibility of drugs and procedures with pregnancy. Treatment must be carried out in coordination with a multidisciplinary team including a gynecologist. We have used hygienic-dietary measures, laxatives, analgesics, and local topicals as therapeutic means. The same observations are made in the literature [7,15]. In the literature, instrumental treatments and surgery will have to be re-discussed at a distance from childbirth, depending on the usual indications [17-20]. First-line treatment of anal incontinence combines specific perineal re-education of the anus with dietary measures and drug prescriptions to regulate intestinal transit Holzheimer *et al*., Dembélé BT *et al*. [19,20]. During pregnancy counseling, 40 women agreed to undergo perineal reeducation after childbirth; during the post-partum period, most of them cited a lack of time, hence the low rate of reeducation. It is therefore vital to inform mothers-to-be of the benefits of post-partum reeducation, whatever the weight of the child and the mode of delivery.

We conducted a prospective study in 10 centers with 1,422 pregnant women. Physicians were pre-trained, and data were processed and analyzed by a single team. This work is the first of its kind in Mali. Well-systematized clinical examinations and anoscope, which complemented those performed, when necessary, enabled us to make precise and detailed diagnoses. Follow-up enabled us to identify treatment compliance and efficacy. Given the lack of frequency in our hospitals, we were unable to calculate the sample size.

## Conclusion

Anal pathologies were common during pregnancy and in the postpartum period affecting over 38% of women in pregnancy and postpartum period. The clinical signs are unpleasant and poorly tolerated by pregnant women. The risk factors are different depending on the pathologies. Well-conducted medical treatment can provide relief to patients. Screening for anal pathologies must be an integral part of prenatal and postnatal consultations.

### What is known about this topic

- *Frequencies of anal pathologies in pregnancy in the words;*
- *Treatment during pregnancy is medical and after delivery, it can be completed*.

### What this study adds

- *The frequency of anal pathologies in pregnancy in Bamako is 38.4%;*
- *The medical treatment of those diseases during pregnancy is available and efficacy;*
- *It is therefore vital to inform mothers-to-be of the benefits of post-partum reeducation, whatever the weight of the child and the mode of delivery*.
